# Practice Patterns and Learning Curve in Transoral Endoscopic Thyroidectomy Vestibular Approach With Neuromonitoring

**DOI:** 10.3389/fendo.2021.744359

**Published:** 2021-10-21

**Authors:** Ting-Chun Kuo, Quan-Yang Duh, Yi-Chia Wang, Chieh-Wen Lai, Kuen-Yuan Chen, Ming-Tsan Lin, Ming-Hsun Wu

**Affiliations:** ^1^ Department of Surgery, National Taiwan University Hospital, Taipei, Taiwan; ^2^ Department of Traumatology, National Taiwan University Hospital, Taipei, Taiwan; ^3^ Department of Surgery, Section of Endocrine Surgery, University of California San Francisco, San Francisco, CA, United States; ^4^ Department of Anesthesiology, National Taiwan University Hospital, Taipei, Taiwan; ^5^ Department of Surgery, Buddhist Tzu Chi General Hospital, Taipei, Taiwan

**Keywords:** endoscopic thyroidectomy, neuromonitoring, thyroidectomy, learning curve, thyroid nodule, thyroid cancer

## Abstract

**Objectives:**

Intraoperative neuromonitoring has not been routinely applied in early experience with the transoral endoscopic thyroidectomy vestibular approach (TOETVA). Because the preparation and surgical interventions are much different from conventional thyroidectomies, most endocrine surgeons willing to adapt to TOETVA lack access to information regarding the practice pattern and proficiency in the learning curve. We aimed to investigate the outcomes and to define the learning curve for TOETVA in this study.

**Methods:**

A retrospective analysis was used on patients who underwent TOETVA at our hospital between December 2016 and July 2019. The cumulative sum graphic model was used to implement the learning curve as a surrogate for procedural proficiency.

**Results:**

The 119 patients had a mean age of 44.65 years and a mean body mass index of 22.49 k/m^2^, including 107 women, 20 thyroiditis, and 106 hemithyroidectomy. The learning curve revealed two phases, an initial (35 cases) and a mature (84 cases) phase, for surgeons based on operation time (144.2 *vs*. 114.2 min, *p* = 0.0001). There were more bilateral thyroidectomies (15.5% *vs*. 0, *p* = 0.0100), larger indicated nodules (6.06 cm^3^ *vs*. 3.32 cm^3^, *p* = 0.0468), or larger thyroids to resect (16.38 cm^3^ *vs*. 8.75 cm^3^, *p* = 0.0001) in the mature phase. Procedure-related complications decreased significantly in the mature phase in comparison to the initial phase (3.57% *vs*. 31.43%, *p* = 0.0001).

**Conclusions:**

The learning curve of TOETVA with neuromonitoring is 35 cases. With the accumulation of proficiency, the indications will expand. Step-by-step improvements from the experience of each case can reduce procedure-related complications.

## Introduction

Many different surgical techniques to minimize or hide neck scars during thyroidectomies exist (1, 2). A new concept is the natural orifice transluminal endoscopic surgery (3). Early experience indicates that the safety profile and outcomes of the transoral endoscopic thyroidectomy vestibular approach (TOETVA) are satisfactory (4–8). Intraoperative neuromonitoring (IONM) and visually identifying the recurrent laryngeal nerve (RLN) during thyroid surgery have gained widespread acceptance as the gold standard (9, 10). Recently*, IONM* has been applied to TOETVA and is performed according to the standards established by the International Neural Monitoring Study Group (INMSG) Guidelines (9, 11). However, IONM has not been widely employed in previous TOETVA studies.

Establishing practice patterns and learning curve proficiency for TOETVA with neuromonitoring can provide a guide for development. The cumulative sum (CUSUM), a sequential analysis technique, has been well utilized in studying learning curves with a graphic model (12). Few studies have evaluated the learning curve for TOETVA, some of which were limited by their diverse surgical methods (*with* and without *neuromonitoring*), in combination with remote access of the robotic systems or endoscopies, and their small sample sizes (13–15). Moreover, for TOETVA with neuromonitoring, the preparation and the surgery itself are much different from a conventional thyroidectomy. Most endocrine surgeons who are willing to adapt to TOETVA with neuromonitoring have a lack of access to information regarding the expected learning curve.

As we had adapted neuromonitoring system for TOETVA from the first case, we aimed to investigate the short-term outcomes and to define the learning curve for this procedure.

## Materials and Methods

### Patient Eligibility and Study Design

This study had a retrospective design and was approved by the institution’s supervisory committee with waiver of informed consent for the utilizations of retrospective hospital data by the National Taiwan University Hospital Research Ethics Committee (NTUH REC approval umber: 201906051RINA) following International Conference on Harmonization of Guidelines for Good Clinical Practice. The medical records of all patients who underwent TOETVA procedures between December 2016 and July 2019 at NTUH were reviewed, and the patients were consecutively enrolled in this study. All operations were performed by a single main surgeon (M-HW), and an anesthesiologist (Y-CW) and other assistant surgeons helped as one surgical team. The surgical team had experience in more than 2,000 cases of open thyroidectomy and 300 cases of minimally invasive thyroidectomy with/without video assistance (MIVAT) in the last 8 years. The surgical and anesthesia care team adapted IONM in 157 cases per year since 2014. Patients’ thyroid function, thyroid sonography, and fine needle aspiration cytology were evaluated preoperatively. Computed tomography was conducted in selected cases.

### Anesthesia and Preparation

For TOETVA with neuromonitoring, either a standard nasotracheal or an alternative orotracheal tube with IONM can be used. In our setting, for better operative field exposure and disinfection concerns, all patients received neuromonitoring using either the NIM 3.0 EMG Nerve Monitoring System (Medtronic, Jacksonville, Florida, USA) or the ISIS IOM System (Inomed Medizintechnik GmbH, Hausgrün, Emmendingen, Germany) through nasoendotracheal intubation.

Remifentanil, a short-acting opioid, was used for target-controlled infusion for pain relief and sedation with dose titration according to the heart rate and blood pressure. Nimbex (cisatracurium besylate), a non-depolarized muscle relaxant, was given depending on the monitoring of the neuromuscular block to an ideal Train-of-Four ratio of 40% (TOF Watch SX^®^ monitor) (16) to facilitate nerve identification and smooth INOM intraoperatively.

### Surgical Technique and Neural Integrity Monitoring

We used the same procedure as that used by Angkoon Anuwong (4, 6). We utilized the IONM steps recommended by the INMSG guidelines for all cases (9). A disposable 230-mm-long ball-tip monopolar stimulation probe (PSP1002, Medtronic, Jacksonville, Florida, USA) was used for nerve stimulation. Intraoperative signal loss and the mechanism of RLN injury were recorded.

Details of postoperative management were the same as those described by Angkoon Anuwong (6, 17). All patients were scheduled to be discharged on postoperative day (POD) 2.

### Outcome Evaluation

Regarding time, the total time (TT) includes the preparation time (PT) and the operation time (OT). TT was the time interval in which the patient was in the operation room, and PT was defined as the period including anesthesia, outfit positioning, disinfection, and draping.

Any visible skin injuries were evaluated and photographic evidence was recorded. Preoperative and postoperative vocal cord movements were evaluated with ultrasound. If there were suspect symptoms, direct laryngoscope evaluation was performed. Transient RLN palsy was defined as vocal cord movement recovered within 6 months postoperatively.

### Learning Curve Analysis

To assess the progress of TOETVA with neuromonitoring in our study, the TT, PT, and OT of each patient were documented chronologically, from the first case to the last case. The CUSUM_OT_ of the first patient was the difference between the value of the first patient and the mean value of all enrolled patients in terms of OT. Subsequently, the CUSUM_OT_ of the included patients was in accordance to that of the previous procedures. The CUSUM graphic model was used to implement the learning curve in our series as a surrogate for procedural proficiency (15). In addition, risk-adjusted CUSUM (RA-CUSUM) methods represented the observed TOETVA procedure-related complications and was calculated from the logistic regression model as risk factors (18).

### Statistical Analysis

The data were summarized by descriptive statistics. Categorical variables are shown as numbers and percentages, while continuous variables are displayed as means and standard deviations. Patient characteristics were analyzed with a Student’s *t*-test or analysis of variance for continuous variables and with a chi-square test or Fisher’s exact test for categorical variables. The statistical analyses were performed using SAS 9.4 (Cary, NC, USA). All *p*-values were two sided and the significance level was set at 5%.

## Results

Among the consecutive 119 patients scheduled to undergo TOETVA with neuromonitoring, all were assessed for eligibility and completed the intended procedure. The enrolled patients had a mean age of 44.65 ± 12.27 years old, with a mean body height of 160.66 ± 6.67 cm, a mean body weight of 58.36 ± 9.75 kg, and a mean body mass index of 22.49 ± 3.28 kg/m^2^. Of these, 107 (89.9%) were women; 106 (89.1%) received a hemithyroidectomy, with 64 (60.4%) right-sided procedures and 20 (16.8%) patients had thyroiditis. The diagnosis revealed 44 (37.0%) nodular goiters, 33 (27.7%) follicular adenomas, 39 (32.8%) papillary thyroid carcinomas (PTCs), and 3 (2.5%) follicular thyroid carcinomas (FTCs). All patients completed follow-up for at least 6 months, and there was no missing outcome data. The baseline characteristics and surgical outcomes of the cohort are presented in **Table 1**.

**Table 1:** Demographic data and operative details of the 119 study patients.

Variable	
Age, mean (SD), years	44.65 (12.27)
Sex, male/female	12/107
Thyroiditis	20
Bilateral thyroidectomy/hemithyroidectomy	13/106
Thyroid disease	
Nodular goiter	44
Follicular adenoma	33
Papillary thyroid carcinoma	39
Follicular thyroid carcinoma	3
Indicated nodule volume, mean (SD), cm^3^	5.25 (6.86)
Thyroid to be resected volume, mean (SD), cm^3^	14.13 (8.97)

Values are presented as mean/(standard deviation, SD).

### Learning Curve Analysis

**Figure 1:**
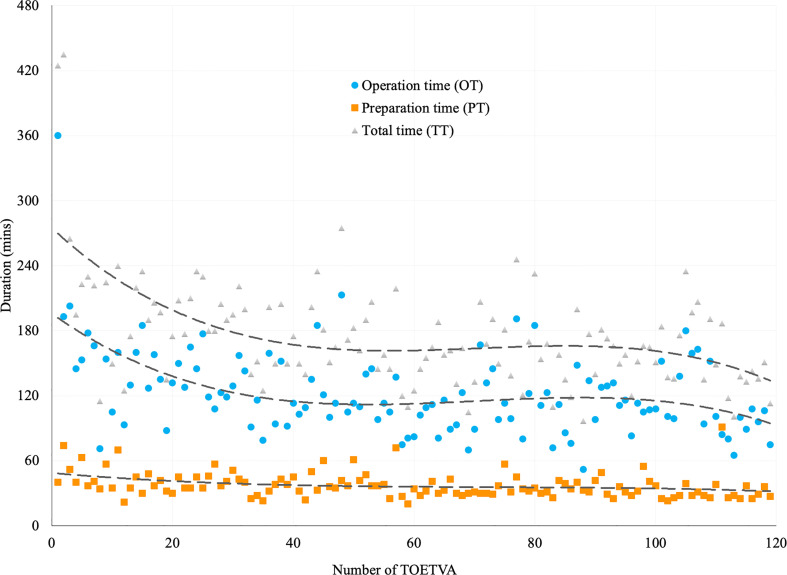
Trend lines for the duration of TOETVA using power cubic equation method. Duration of total time (TT, in gray triangles), preparation time (PT, in orange squares), and operation time (OT, in blue dots) in 119 patients receiving TOETVA.

The average TT, PT, and OT were 175.4 ± 50.3, 37.5 ± 11.6, and 123.0 ± 39.6 min, respectively. The trend lines of both TT and OT showed obvious slopes in time expense during the initial cases (**Figure 1**).

**Figure 2:**
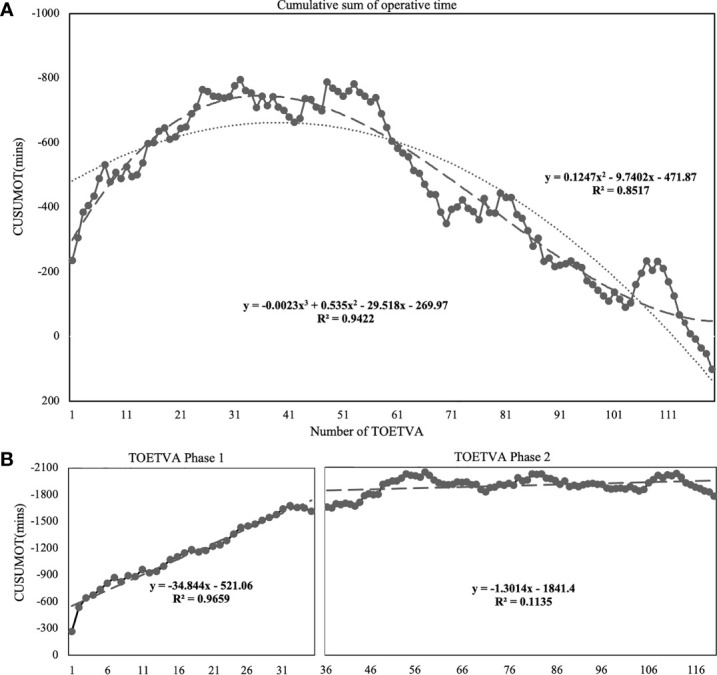
Learning curve for TOETVA using cumulative sum (CUSUM) method. **(A)** CUSUM of operation time plotted against number of TOETVA (solid line with marked point). The dashed line represents the curve of best fit for the plot as a third-order polynomial using the equation CUSUM_OT_ = −0.0023 × patient number^3^ + 0.535 × patient number^2^ − 29.518 × patient number − 269.97, *R*² = 0.9422. The dotted line represents the curve using a second-order polynomial using the equation CUSUM_OT_ = 0.1247 × patient number^2^ − 9.7402 × patient number − 471.87, *R*² = 0.8517. **(B)** According to the change in the slope, the cutoff points of the two phases were best calculated as 35.90 (third-order polynomial). Lines of the best fit for the two phases of the CUSUM_OT_ learning curves included 35 and 84 cases, respectively.

The CUSUM_OT_ learning curve was best modeled as a third-order polynomial equation with a higher *R* ^2^ value of 0.9422 as follows: CUSUM_OT_ (in minutes) = −0.0023 × patient number^3^ + 0.535 × patient number^2^ − 29.518 × patient number − 269.97 (**Figure 2A**). According to the change in the slope, two distinct phases were illustrated in **Figure 2B**. The first 35 cases were included in the initial phase (Phase 1) and the next 84 cases were included in the mature phase (Phase 2).

Additionally, RA-CUSUM learning curve represented the observed TOETVA procedure-related complication from the logistic regression model as risk factors of phase 1, and bilateral procedure, obesity, and malignancy (**Supplementary File**) revealed the first phase at 35 cases.

### Outcomes and Clinical Differences Between Phases Defined Using CUSUM Learning Curve Analysis

#### Preparation

Nasotracheal intubation was completed in 117 patients and 2 patients received orotracheal intubation due to both tube cuff disinflation and air leaks caused by nasal septum deviation during tube insertion. The average intubation depth, from the nostril to the tip of the tube, was 26.0 ± 0.6 cm in nasal intubated cases. Nerve integrity stimulation was achieved in all patients and 132 nerves at risk. There were five cases of preparation-related incidences that included two cases of facial indentations over the ala nasi resulting from nasotracheal tubes compressive injury and three cases of nasal mucosal bleeding from intubation trauma. These were in the early seven cases.

#### Operation

There were five thyroidectomy-related complications. Transient vocal cord palsy was noted in two cases (2/119) whose nerve injuries were indicated by the loss of IONM signals after applying clips for the bleeding of a vessel near RLN. The findings were also confirmed with postoperative laryngoscopy examination. Transient hypoparathyroidism was observed in three patients.

There were 14 procedure-related complications: two cases of prominent subcutaneous emphysema, one focal infection, three cases of transient chin numbness, four cases of neck ecchymosis (resulting from dissection in the neck region), and four cases of oral commissure erosion (resulting from collision of endoscopic instruments at the edge of mouth).

Details of the surgical outcomes between phases are shown in **Table 2**. There was no significant difference in massive intraoperative blood loss, postoperative visual analog scale (VAS) pain scores, hospitalization, thyroidectomy-related complications, and/or overall complications between the two phases. There were significantly higher procedure-related complications in Phase 1 (31.4% *vs*. 3.6%, *p* = 0.0001, **Table 2**).

**Table 2:** Comparisons of demographic data and surgical outcomes between phases.

	Phase 1 (*n* = 35)	Phase 2 (*n* = 84)	*p*-value
Age, mean (SD), years	42.47 (10.80)	45.56 (12.78)	0.2128
Sex, male/female	4/31	8/76	0.7461^F^
Body mass index, mean (SD), kg/m^2^	22.51 (3.99)	22.48 (2.95)	0.9628
Thyroiditis	7	13	0.5476
Operative time, mean (SD), min	144.15 (49.43)	114.23 (31.06)	0.0001*
Bilateral thyroidectomy/hemithyroidectomy	0/35	13/71	0.0100*^F^
Hemithyroidectomy (right/left)	35 (19/16)	71 (45/26)	0.4485
With central neck dissection in cancer patients	2	7	0.6925
Massive blood loss	2	2	0.5800^F^
Hospitalization, mean (SD), day	1.9 (0.5)	2.0 (0.4)	0.5308
Conversion or reoperation	0	0	
Overall complications	12	8	0.0010*
Thyroidectomy-related	0	5	0.3198^F^
RLN palsy (transient/permeant)	0/0	2/0	1^F^
Hypoparathyroidism (transient/permeant)	0/0	3/0	0.5544^F^
Hematoma or seroma	0	0	-
Procedure-related^†^	11	3	0.0001*
Subcutaneous emphysema or pneumomediastinum	2	0	0.0847^F^
Focal infection	0	1	1.0000^F^
Chin numbness	3	0	0.0239*^F^
Neck ecchymosis	4	0	0.0060*^F^
Oral commissure erosion	2	2	0.5800^F^
Thyroid disease, benign/malignancy	21/14	56/28	0.4881
Indicated nodule volume, mean (SD), cm^3^	3.32 (4.32)	6.06 (7.55)	0.0468*
Thyroid to be resected volume, mean (SD), cm^3^	8.75 (4.93)	16.38 (9.33)	<0.0001*

*Statistical significance, p < .05. Scheffe post hoc was used. ^F^Fisher’s exact test. Values are presented as mean/(standard deviation, SD). RLN, recurrent laryngeal nerve.

^†^One case in phase 1 had subcutaneous emphysema and chin numbness.

## Discussion

This study aimed to investigate the short-term outcomes and to define the learning curve for those adapting the neuromonitor system to TOETVA. We illustrated detailed components related to the standardized preparation and the surgery elements including the TOETVA procedure and thyroidectomy in a team-based approach.

The concept of the experimental procedure “natural orifice transluminal endoscopic surgery (NOTES)” was first presented in 2000 (19) and was applied to the neck in 2008 (20). TOETVA furnished a scarless choice for thyroidectomy after its release by Anuwong in 2016 (4) with excellent outcomes. TOETVA has become increasingly prevalent in recent years (13, 21).

TOETVA minimizes the need for tunneling flap dissection when compared with other extra cervical approaches (anterior chest wall, breast, or axillary approaches) (13, 21, 22). The surgical techniques and overall complication rates, whether thyroidectomy or procedure-related, of TOETVA were regarded as practical and appropriate (13, 14). However, the TOETVA technique relies on only three ports in a limited oral-vestibular space and the direction of the view is opposite. Beginners may encounter problems in handling difficult situations (6). In addition, applying a neuromonitoring system to TOETVA requires modification, including the preparation and the surgery, and the possible complications remain unaddressed. Therefore, a learning curve would exist for any team new to this procedure irrespective of the surgeon/anesthesia’s extent of prior experience in traditional open or laparoscopic thyroidectomies.

The CUSUM analysis in our series was initially designed to monitor the performance of the industrial sector. It has been applied to surgical technique learning curves, which can be updated following each procedure (12, 23, 24). The learning curve varied with the various approaches to thyroidectomies. For endoscopic thyroidectomy, 25 to 60 cases in the primary stage have been reported to use the breast approach (25) to achieve decreased operation time and complications. Christopher reported the learning curve of TOETVA for 11 cases using a simple moving average (SMA) of order 3 (15). However, bilateral thyroidectomy was excluded, and the number of cases (*n* = 30) was relatively small. The learning curve for our TOETVA with neuromonitoring was reported for 35 cases. The abundant sample size in the study reflects more of the real process. The series was performed by a single surgical team, executing conventional thyroidectomies. We began TOETVA and had limited experience performing thyroidectomies with other remote access approaches. Prior experience with robotic or endoscopic extra cervical approaches may refine the skill proficiency in the TOETVA build-up period. Camenzuli et al. reported that the neuromonitoring system is not routinely used in all patients for TOETVA and IONM was used in four studies for their initial experience but no more than 20 cases (Christian Camenzuli, 2018). Of the largest series, Anuwong mentioned an estimated learning curve of 7–10 cases for TOETVA with neither routinely used IONM nor detailed statistical analysis (6). Chai recently reported 58 cases for surgical outcomes and learning curve of TOETVA with routine IONM (26). These meant the true learning should be based on standardized preoperative preparation including anesthesia care, IONM, and TOETVA procedures in a team-based approach with larger models. Although several previous studies evaluated the learning curve, the learning curve of transoral thyroidectomy has not been determined clearly.

In this series, the incidence of procedure-related complications decreased significantly in Phase 2. This means that the operation time was decreased along with the procedure-related complications. Some complications would not have occurred in early cases because the starters always selected perfect cases for new procedures. The indication of patient selection is thought to be expended after surgeons have experience. In the present study, we adopted a stringent policy, and indeed, the proportion of more challenging cases (bilateral procedures and larger thyroid or nodule volume) increased with increasing experience.

It is recommended to begin with simple cases of TOETVA to achieve an optimal learning curve and advance to difficult cases at the end of the learning curve (22). Thus, a large case series is important to demonstrate the true blueprint and to assist the beginners to shorten their operation times and prevent possible complications.

The preparation for neuromonitoring in TOETVA is different from that of conventional thyroid surgery or extra cervical approaches. For TOETVA, the cephalic site of patients is occupied by surgeons and instruments, which makes it impossible for the anesthesiologists to check the neural sensor position or airway condition if necessary. A tube malposition or dislodgement was barely adjustable during the operation; thus, the preparation is assumed to have settled perfectly before operation. In our series, the RLN neural detection rate was 100%. There was no obvious time change in this study in the preparation period. We believe that this may be due to earlier experience of the anesthesiologists who had adopted the preparation in conventional thyroidectomies. The main difference for TOETVA in the preparation steps was in the nasal tracheal intubation and facial area protection. We encountered facial indentations over the ala nasi and nasal mucosal bleeding in the learning period, which is caused by imperfect protection and the long operation time. Preparation with the orotracheal tube was as described in other studies (6, 11, 27), and in our two patients who failed to undergo nasotracheal intubation, it might not necessarily interfere in the surgical field. However, complete disinfection around the tube area remains challenging, and we currently use nasal tracheal intubation.

Both the anatomic and physiologically functional preservation of RLN is important in thyroid surgery. However, procedures are more technically difficult in TOETVA because of the problems of the unusual vector of dissection and the relatively poor visibility due to limited surgical space. The incidence of transient and permanent RLN injury in TOEATVA, without routine confirmed with IONM or post-operative laryngoscopy, was about 4.3% and 0.1%, respectively (13, 14). IONM during TOETVA is reported as feasible and safe in order to identify or monitor the function of the RLN and EBSLN (5, 11, 28). Transient RLN injury occurred in 1.7% of cases and 1.5% of nerves at risk in our series without any permanent RLN injury, which is lower than in previous reports (13, 14, 29, 30). The routine use of IONM in our series has the advantage of demonstrating the real incidence and the mechanism of injury. The correlation between the intraoperative signal loss and the post-operative voice change/laryngoscope findings was 100% in this series, which was compatible with Chiang proposed up to 100% correlation between the EMG signals under standardized IONM and the vocal cord function (31). Transcutaneous laryngeal ultrasonography (TLUSG) has been proposed as a promising noninvasive technique, whose preoperative sensitivity, specificity, and accuracy were 100%, 70.0%, and 70.5%, respectively, for nonoverweight patients, and alternative to flexible fiberoptic laryngoscopy (32). It allowed us to identify problems in our two patients who had signal loss intraoperatively. Therefore, we could rescue the RLN instantly without causing irreversible damage. In addition, no RLN injury occurred in the leaning phase in our series, which may further emphasize the importance of IONM use especially when working to establish a new procedure.

The study is limited by a retrospective design. The study may simplify the variation between surgeons and anesthesiologists as all cases in our series were performed by the same team.

## Conclusion

TOETVA with neuromonitoring requires a team to accumulate experience with equipment and troubleshooting algorithms to achieve safety and to determine the feasibility of the procedure. Endocrine surgeons without previous experience in other endoscopic thyroid surgeries could perform stable procedures and overcome the learning curve after the initial phase of 35 cases. Our experience may allow medical centers that are introducing the TOETVA with neuromonitoring and building their teams to move through the learning curve as safely as possible.

## Data Availability Statement

The original contributions presented in the study are included in the article/**Supplementary Material**. Further inquiries can be directed to the corresponding author.

## Ethics Statement

The studies involving human participants were reviewed and approved by 201906051RINA. Written informed consent for participation was not required for this study in accordance with the national legislation and the institutional requirements.

## Author Contributions

T-CK, M-HW, K-YC, C-WL, and Y-CW proposed the study. T-CK, M-HW, and K-YC performed the research, analyzed the data, and wrote the first draft. T-CK, M-HW, and Y-CW collected the data. T-CK, Q-YD, M-HW, C-WL, and M-TL analyzed the data. All authors contributed to the design and interpretation of the study and to improvement of the manuscript drafts. All authors have read and agreed to the published version of the manuscript.

## Funding

This study was supported by the National Taiwan University Hospital, Taiwan (110- S5007) and Ministry of Science and Technology, Taiwan (110-2314-B-002-047)

## Conflict of Interest

The authors declare that the research was conducted in the absence of any commercial or financial relationships that could be construed as a potential conflict of interest.

## Publisher’s Note

All claims expressed in this article are solely those of the authors and do not necessarily represent those of their affiliated organizations, or those of the publisher, the editors and the reviewers. Any product that may be evaluated in this article, or claim that may be made by its manufacturer, is not guaranteed or endorsed by the publisher.
