# Supplementary figures and images for: Practice Patterns and Learning Curve in Transoral Endoscopic Thyroidectomy Vestibular Approach With Neuromonitoring
Source: Front Endocrinol (Lausanne). 2021 Oct 21;12:744359. doi: 10.3389/fendo.2021.744359 (PMC8594485; doi:10.3389/fendo.2021.744359)

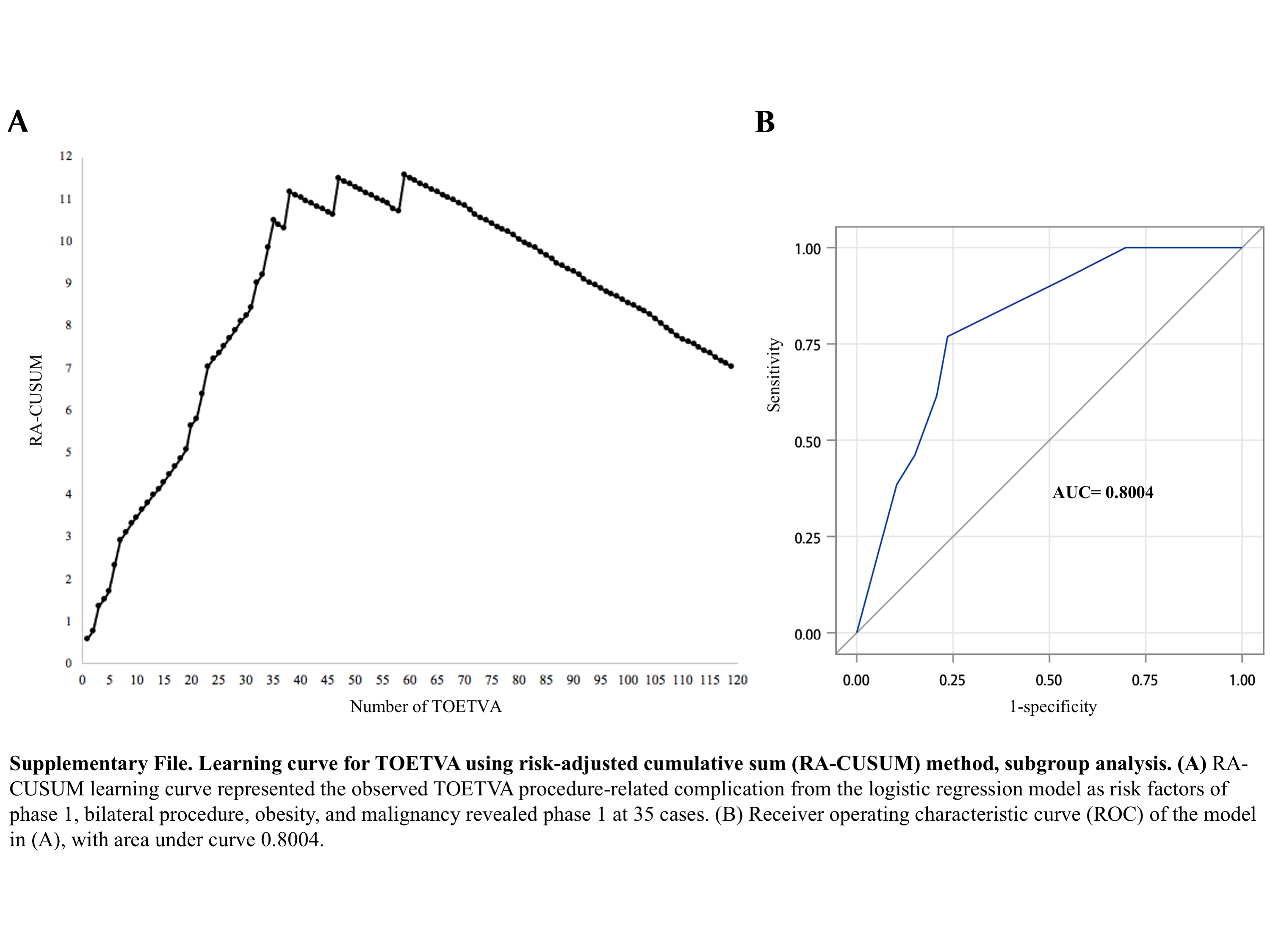

Supplement: Supplementary file 1 [file Image_1.tiff]
